# SNPAAMapper-Python: A highly efficient genome-wide SNP variant analysis pipeline for Next-Generation Sequencing data

**DOI:** 10.3389/frai.2022.991733

**Published:** 2022-09-12

**Authors:** Chang Li, Kevin Ma, Nicole Xu, Chenjian Fu, Andrew He, Xiaoming Liu, Yongsheng Bai

**Affiliations:** ^1^USF Genomics and College of Public Health, University of South Florida, Tampa, FL, United States; ^2^Canyon Crest Academy, San Diego, CA, United States; ^3^Obra D. Tompkins High School, Katy, TX, United States; ^4^College of Arts and Sciences, Kent State University, Kent, OH, United States; ^5^Pittsford Mendon High School, Pittsford, NY, United States; ^6^Next-Gen Intelligent Science Training, Ann Arbor, MI, United States; ^7^Department of Biology Eastern Michigan University, Ypsilanti, MI, United States

**Keywords:** Next-Generation Sequencing, SNP, python, mutation, variant annotation, pipeline

## Abstract

Currently, there are many publicly available Next Generation Sequencing tools developed for variant annotation and classification. However, as modern sequencing technology produces more and more sequencing data, a more efficient analysis program is desired, especially for variant analysis. In this study, we updated SNPAAMapper, a variant annotation pipeline by converting perl codes to python for generating annotation output with an improved computational efficiency and updated information for broader applicability. The new pipeline written in Python can classify variants by region (Coding Sequence, Untranslated Regions, upstream, downstream, intron), predict amino acid change type (missense, nonsense, etc.), and prioritize mutation effects (e.g., synonymous > non-synonymous) while being faster and more efficient. Our new pipeline works in five steps. First, exon annotation files are generated. Next, the exon annotation files are processed, and gene mapping and feature information files are produced. Afterward, the python scrips classify the variants based on genomic regions and predict the amino acid change category. Lastly, another python script prioritizes and ranks the mutation effects of variants to output the result file. The Python version of SNPAAMapper accomplished the overall speed by running most annotation steps in a substantially shorter time. The Python script can classify variants by region in 53 s compared to 166 s for the Perl script in a test sample run on a Latitude 7480 Desktop computer with 8GB RAM and an Intel Core i5-6300 CPU @ 2.4Ghz. Steps of predicting amino acid change type and prioritizing mutation effects of variants were executed within 1 s for both pipelines. SNPAAMapper-Python was developed and tested on the ClinVar database, a NCBI database of information on genomic variation and its relationship to human health. We believe our developed Python version of SNPAAMapper variant annotation pipeline will benefit the community by elucidating the variant consequence and speed up the discovery of causative genetic variants through whole genome/exome sequencing. Source codes, test data files, instructions, and further explanations are available on the web at https://github.com/BaiLab/SNPAAMapper-Python.

## Introduction

Next-Generation Sequencing is a technique to rapidly sequence a genome and was developed because of the Human Genome Project, which successfully sequenced a human genome over a period of 23 years (www.genome.gov/human-genome-project) and cost around $2.7 billion in 1991 Fiscal Year Dollars, equivalent to $5.6 Billion 2022 Fiscal Year Dollars. Today, a human genome can be accurately sequenced for as low as $600 (Preston et al., 2022). In 2013, sequencing a whole human genome took between 1 and 2 days (Lewis, 2013).

With the decreasing cost and increasing availability of the Next-Generation-Sequencing technique (Barba et al., 2014), our ability to discover variants in the human genome has been revolutionized. More variants have been reported and discovered. Our ability to interpret or annotate these variants becomes a major gap in effectively using genomics data in understanding diseases. To address this issue, multiple variant annotation tools that locate and assign information about variants have been developed.

One such tool is SNPAAMapper, a variant analysis tool developed in 2013 in the Perl coding language. SNPAAMapper contains two general algorithms: one that generates annotation tables with coding and other information annotated for each exon, and one that reads the generated annotation tables and assigns identified variants to the genomic loci and classifies them by region (Bai and Cavalcoli, 2013).

The original SNPAAMapper used the Perl coding language to make alignment of input DNA sequences, which may have sub-optimal performance. This inefficiency and inability to handle big data in a timely manner placed a hurdle in its wide applications. In this study, we chose to update and modify SNPAAMapper to substantially increase the speed of the program to fulfill the current need of the genomics field. Additionally, we presented an improved output to facilitate downstream data processing and analysis.

## Methods

### Input data acquisition

The reference genomes used in the paper were sourced from the UCSC genome browser (https://genome.ucsc.edu/). The UCSC Genome Browser is a web-based tool that allows researchers to example all 23 chromosomes of the human genome all the way down to an individual nucleotide. It also contains data on the genomes of more than a 100 other organisms. The genome browser was created and maintained by Jim Kent and David Haussler at UCSC in 2000 as a resource for the distribution of results from the Human Genome Project. It was funded by the Howard Hughes Medical Institute and the National Human Genome Research Institute (NHGRI) (https://genome.ucsc.edu/goldenPath/history.html).

When testing our tool, we used both a small test dataset (number of variants = 80) and data file from the ClinVar database (https://www.ncbi.nlm.nih.gov/clinvar/) (number of variants = 1,440,883), a publicly accessible archive of reports of relationships between human variations and phenotypes. ClinVar is crowdsourced and relies on the submission of reports by researchers and clinical labs. The default format for ClinVar database is VCF and the database file was downloaded in assembly GRCh37/hg19 for the human reference genome on May 28, 2022. VCF is the default format for ClinVar to store and report variants, including point mutations and short insertions/deletions. In the “INFO” columns, some related annotation information was also provided by ClinVar, such as the associated clinical significance, associated diseases, gene annotation etc.

### Algorithm description

**Figure 1:**
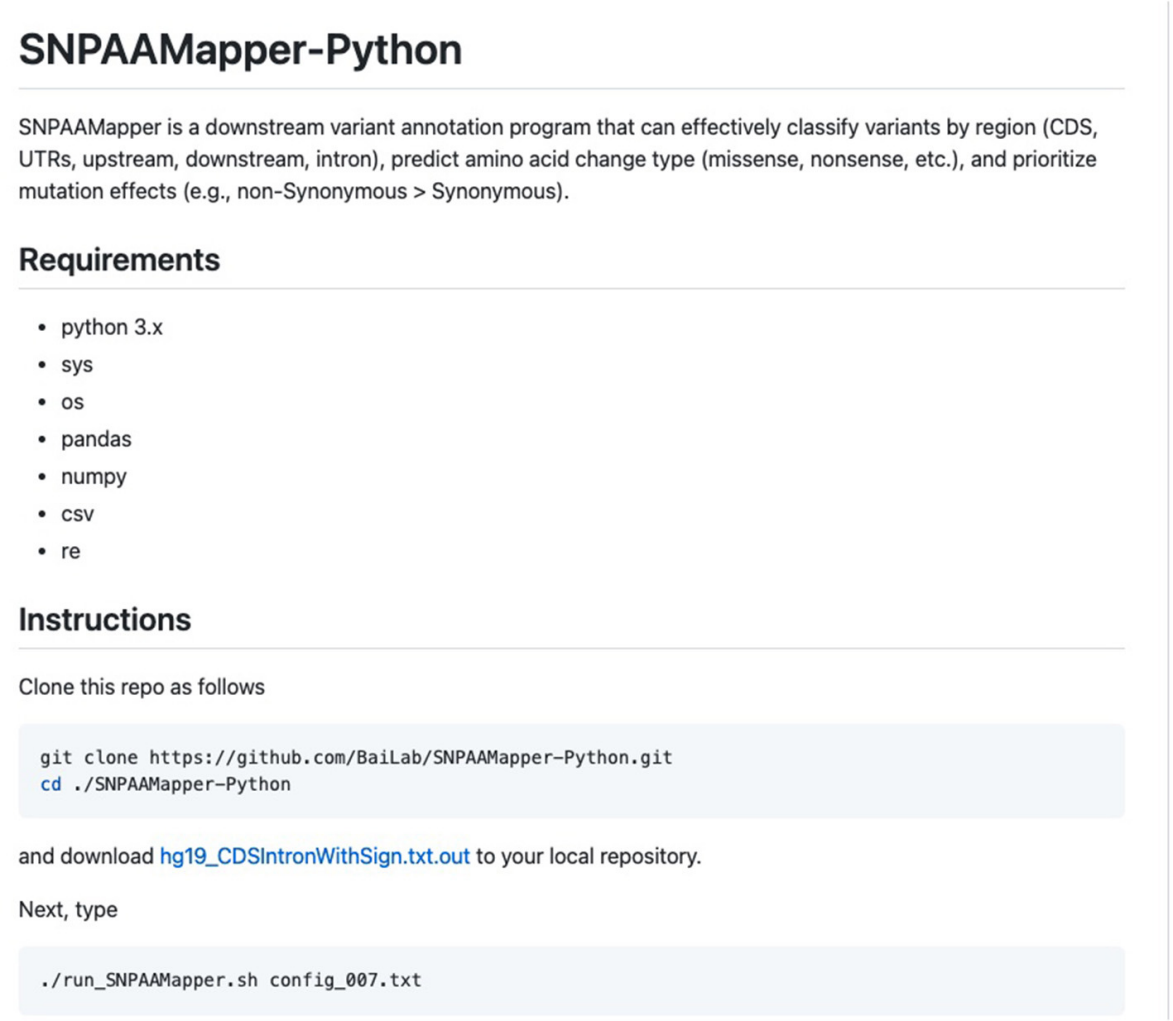
Screenshot of the GitHub website of SNPAAMapper-Python.

The pseudocodes for SNPAAMapper-Python algorithms are described in Algorithm 1 (see Supplementary materials). There are two modules of the algorithm: (1) Preprocess the gene structure to build annotation for each exon; (2) Map identified variants onto the genomic location and report the hit class. In the Python version of SNPAAMapper, the second script for processing exon annotation files and generating feature start and gene mapping files performs extremely better than the one in the original Perl version. The screenshot for SNPAAMapper on the GitHub site is shown in Figure 1.

### Usage

As the input, a VCF file is required for annotation. There are two methods to use the program, an end-to-end option and a step-by-step option. For the end-to-end option, users can use the *config.txt* file to configure the running parameters and define input files. The running parameters are “vcfFile = clinvar_20220528.vcf, intronBoundary = 6, geneAnnotation = ChrAll_knownGene.txt, conversionFile = kgXref.txt, sequenceFile = hg19_CDSIntronWithSign.txt.out.” Then users can use command.*/run_SNPAAMapper-Python.sh config.txt* to generate the final output by running through each step automatically. This option is recommended for all users by default. For the step-by-step option, the users will have to run through the Python scripts step-by-step in the following orders: (1) Generate exon annotation file; (2) Process exon annotation files and generate feature start and gene mapping files (*Algorithm_preprocessing_exon_annotation_RR.py*); (3) Classify variants by regions (CDS, Upstream, Downstream Intron, UTRs...) (*Algorithm_mapping_variants_reporting_class_intronLocation_updown.py*); 4() Predict amino acid change type (*Algorithm_predicting_full_AA_change_samtools_updown.py*); (5) Prioritize mutation effects (*Algorithm_prioritizing_mutation_headerTop_updown.py*). This option is recommended for more advanced users and for users who are only interested in the intermediate outputs. The final output will be an annotated variant file, with each row representing a unique input variant and each column representing one piece of annotated information.

## Results

### Annotated file

For each variant in the input VCF file called by SAMTools (Li et al., 2009), there is a corresponding row in the output annotated file. The final output will be an annotated variant file, with each row representing a unique input variant and each column representing one piece of annotated information. Specifically, there 21 columns with unique annotation information. For VCF files containing individual genotype data, the first column specifies the sample ID. The other 20 columns are as follows: “Chromosome,” “Variant Position,” “Gene Symbol,” “UCSC ID,” “Strand,” “AA Position of Mutation (for CDSHIT),” “Variant Type,” “Amino Acid Ref (Codon) -> AA SNP (Codon),” “Variant Class,” “Ref AA chain,” “Alt AA chain,” “Hit Type,” “Known dbSNP,” “Ref nt,” “Alt nt,” “Quality,” “Depth,” “Allele Freq,” “Read Categories,” and “Info.” A table with column descriptions for the first 15 columns of the VCF output file can be found in Supplementary Table 1. The remaining five columns are variant calling information extracted from the VCF output file.

### ClinVar database

ClinVar is a widely used database that links variants to their functional importance (pathogenicity) (Landrum et al., 2015). ClinVar provides a full download of their database in VCF format. Aside from potential phenotype/clinical association information, ClinVar provides some basic annotation for the variants, such as HGVS-nomenclature, types of variants (single nucleotide variant, indels, etc.), and functional consequences (missense, UTR, etc.). While this information is valuable, it is common to expand these annotations for the clinicians/researchers to better understand the functional impact of the variants for the purpose of disease diagnosis, hypothesis-generating/validation.

Currently, ClinVar (20220508) includes 1,440,883 unique variants that are associated with various diseases. It uses a 5-category classification system that groups these variants into pathogenic, likely pathogenic, benign, likely benign, and variant of uncertain significance.

We first compared the running speed of annotating the test file using the original and updated SNPAAMapper (Table 1). We then ran the updated Python version of SNPAAMapper for entire ClinVar database file and found that our updated version was able to generate exon annotation file in 48 s; generate feature start and gene mapping files in 49 s; classify variants by regions in 256 s, It took 124,311 s for predicting amino acid change type. It took 497 s for prioritizing mutation effects. For the Perl version of SNPAAMapper, it takes more than 2 weeks to run all the pipeline steps.

**Table 1:** Speed comparison between original and updated SNPAAMapper for the test dataset.

**Steps**	**Python execution time in seconds**	**Perl execution time in seconds**
Step 1	13	2
Step 2	16	166
Step 3	2	138
Step 4	62	407
Step 5	1	1
Total	94	714

**Figure 2:**
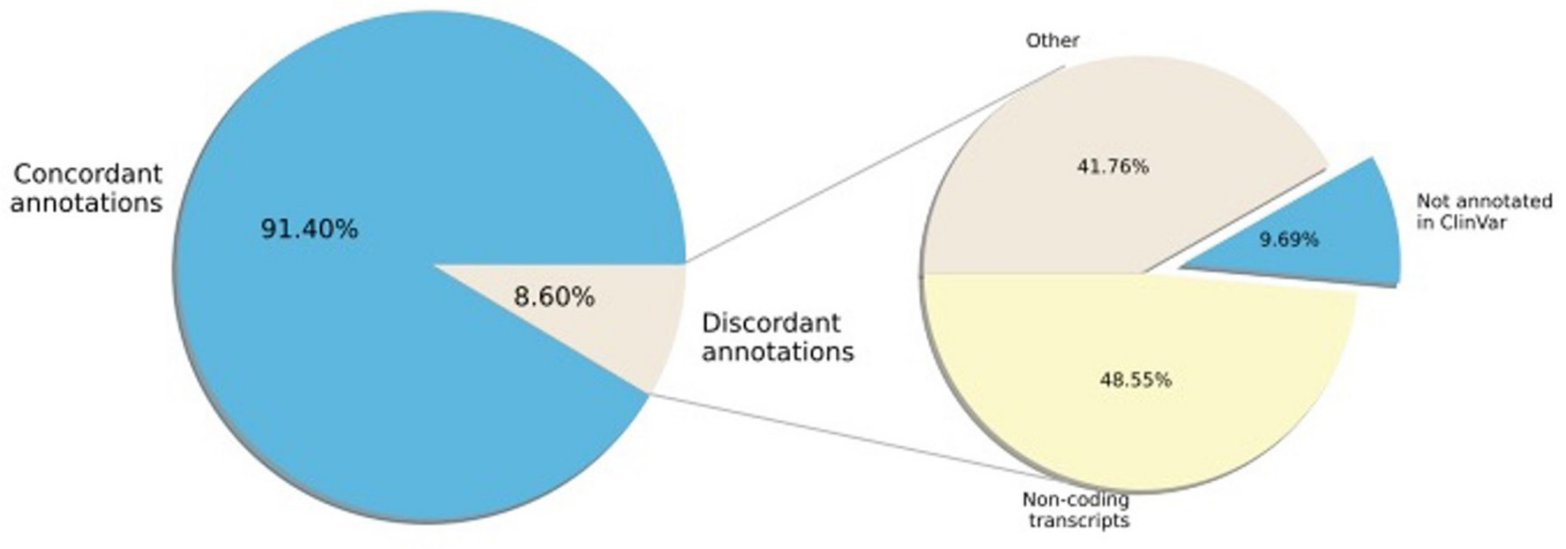
Percentage of ClinVar variant region annotation that are concordant with SNPAAMapper-Python.

**Figure 3:**
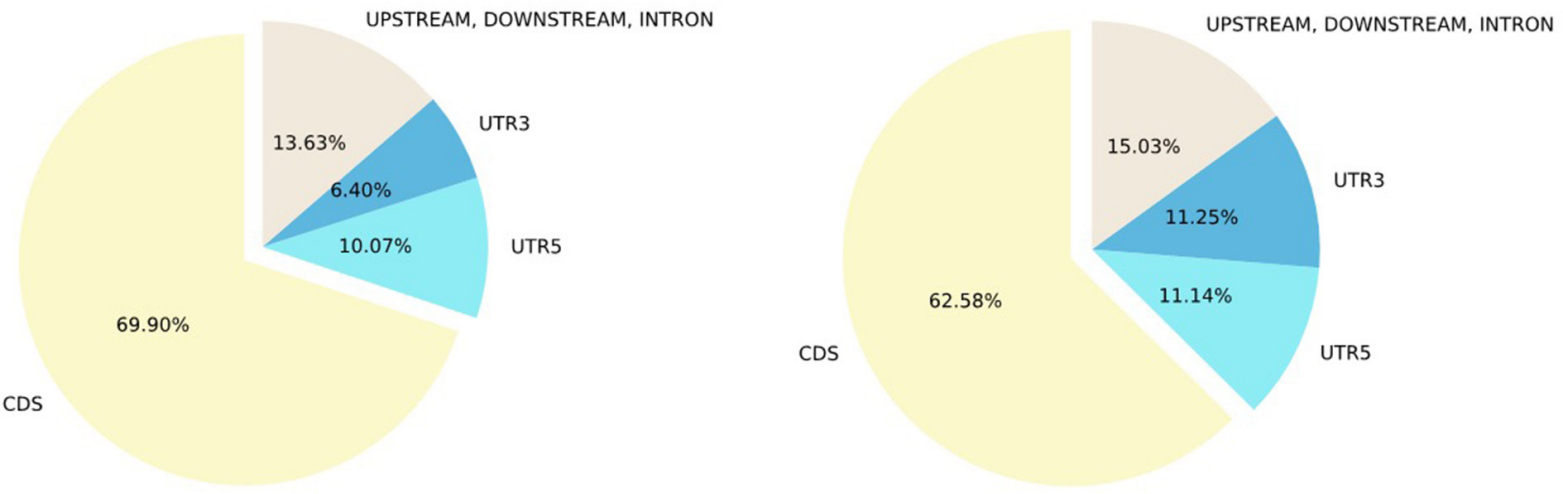
Percentage of ClinVar variants by variant type. Left, Percentage of ClinVar pathogenic variants by variant type; Right, Percentage of ClinVar benign variants by variant region.

Next, we examined the concordance of annotation between ClinVar and SNPAAMapper-Python. We found that 91.4% of the variants have concordant annotations between ClinVar and our tool (Figure 2). Among those variants with discordant annotations, we found that 48.55% of them are annotated as non-coding transcripts variants in ClinVar which was not specifically annotated in SNPAAMapper. Additionally, our tool provided annotation for 10% of the variants that showed no annotation in ClinVar, which highlighted the usefulness of our tool. Importantly, annotations from ClinVar were buried into the “INFO” column with other information, which makes parsing and understanding the information much more difficult, whereas, for our tool, there is a separate column for each specific annotation. Using position-based annotation from SNPAAMapper, we examined the distribution of variants by functional genomics regions (Figure 3). We found that the majority (661,958 for pathogenic variants and 592,638 for benign variants) of reported variants in ClinVar (*n* = 947,008), regardless of their pathogenicity, reside in coding sequences (CDS).

Comparing pathogenic variants (*n* = 103,909) to benign ones (*n* = 340,726), we found that there was a higher percentage of CDS variants and lower percentages of 3'UTR, 5'UTR, and other non-coding sequences for pathogenic variants. This observation illustrated that most of the studies focused on CDS as variants from this region usually have clearer functional consequences. Additionally, using the SNPAAMapper's output, we can easily examine the distribution of variants across different genes. For example, gene TTN has the most unique variants (*n* = 17,915), while 2,448 genes have only 1 unique variant. These observations highlighted our need for investigating the under-studied genes to gain a well-rounded understanding of human genes and genetic mutations. We note that this gain of knowledge is attributable to the easy-to-use format of SNPAAMapper's output.

**Figure 4:**
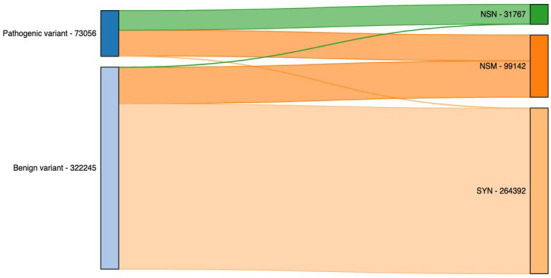
Count of pathogenic and benign variants by their functional annotation. NSN, nonsense; NSM, nonsynonymous; SYN, synonymous.

Finally, we illustrated the importance of including additional exome-specific annotations to help users interpret their data using annotated output from the previous step. First, we looked under the hood for variants residing in CDS. As illustrated in Figure 4, it is not surprising that the vast majority of nonsense (NSN) variants are pathogenic, while most synonymous (SYN) variants are benign. Interestingly, for nonsynonymous (NSM) variants, we observed a similar percentage of the reported pathogenic and benign variants. This highlighted the importance of NSM variants in helping us interpret sequencing variants. As a result, numerous methods have been developed to target NSM variants and predict whether they are functional or not.

**Figure 5:**
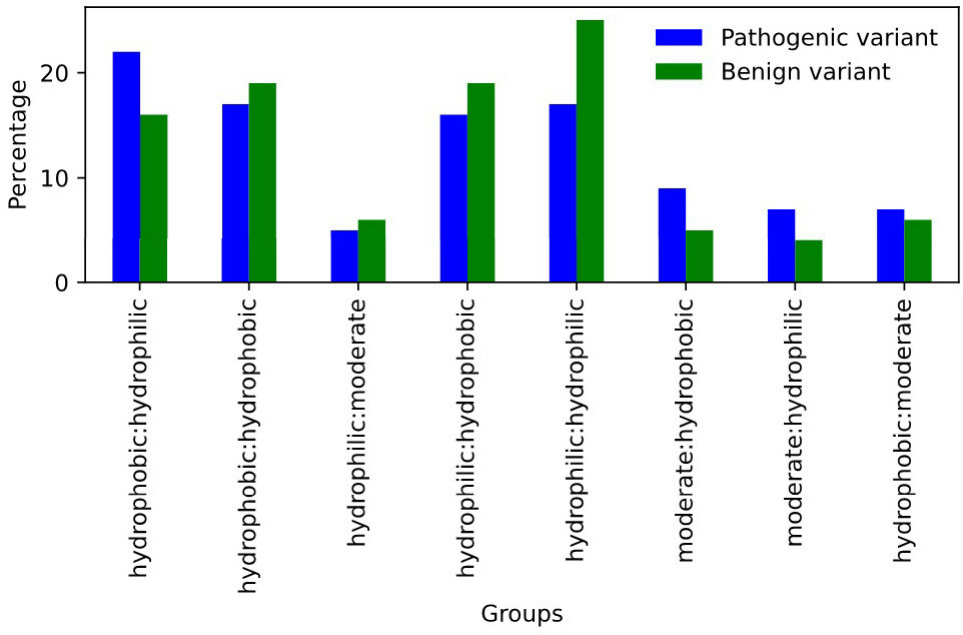
Percentage of variants grouped by their hydropathy.

Another key strength of our SNPAAMapper pipeline was to retrieve the most damaging amino acid variants from genomic variants. This can be used to investigate the property of variants and their impact on the biochemical and physical properties of the amino acid and protein. As illustrated in Figure 5, we plotted the hydropathy of the variants in ClinVar database grouped by their clinical significance. We found that a change in hydropathy was more commonly observed in pathogenic variants. For example, hydrophobic to hydrophilic conversion was substantially enriched in pathogenic variants. On the other hand, benign variants were substantially enriched in variants without pathogenic conversion (hydrophilic to hydrophilic, etc.). This analysis briefly highlighted the importance of providing easy-to-access amino acid variants, as their properties are crucial in understanding the functional consequence of the underlying genomic variants.

### Comparison of performance in run times

We compared the execution time between the original SNPAAMapper and updated SNPAAMapper-Python using the same sample VCF file. The updated program runs significantly faster (8 times) than the original Perl program, with an almost 10-fold increase in speed. This time increase will be substantially more prominent when hundreds or thousands of samples were queried.

## Discussion

The biggest difference between the old tool (SNPAAMapper) and our updated tool (SNPAAMapper-Python) is the change in the programming language. The former runs on Perl, while the latter runs in Python, as the name states. To convert the original Perl pipeline codes, we downloaded and analyzed the original SNPAAMapper code reported in the paper, which was sourced from the previous study (Bai and Cavalcoli, 2013).

Our updated program maintains all the previous features of SNPAAMapper. It grants downstream variant identification and analysis at a record speed. Our tool is self-sufficient and lightweight; external alignment tools and such are not necessary since they are all included in this package. In addition, our tool preserves the original customizability of SNPAAMapper, meaning that it can be easily configured for other species and reference genomes. Another benefit of our program is its greater compatibility; the popularity and use of the Perl programming language are rapidly decreasing (https://www.tiobe.com/tiobe-index/perl/) while the use of Python has been growing at an extreme rate for the last decade (https://www.tiobe.com/tiobe-index/python/). We believe that this upgrade is crucial for researchers due to the impractical run time of the original SNPAAMapper on a test sample (Table 1).

The python version performs more efficiently than the perl version. The reason is that we use an optimized Python-built in module “csv” to read and write tabular data. In particular, the python version is not using any embedded loop as the Perl version by iterating over almost approximately a million rows in the ChrAll_knownGene.txt.exons file for “the number of chromosomes” (about 60) times. Instead, the python version iterates only one time.

We used the same dataset tested in SNPAAMapper for both programs. By running both tools on a Latitude 7480 Desktop computer with 8GB RAM and an Intel Core i5-6300 CPU @ 2.4Ghz, we were able to make an accurate comparison of the execution times for each program. Using this method, we were also able to make time comparisons for each step of both programs.

In addition, we also run SNPAAMapper-python on the ClinVar database file to collect the running statistics. Specifically, we ran the pipeline on an lntel_Core_i7-4770K_CPU@3.5GHz Gentoo Linux box to collect running statistics for ClinVar database file.

Additionally, with the improved output representation, this update enables easy-to-use output where each column represents a single piece of information. These improvements can greatly facilitate downstream analyses and open up opportunities for users to analyze their data using tools like Excel, which is expected to accelerate the translation of information to knowledge. Lastly, our codes are open-sourced and hosted on GitHub, which enables the continuing maintenance, updates and improvements from us and all the users.

We created an end-to-end pipeline with intermediate outputs. The final output is the one that's interesting to most of our users.

To test the ease of use and convenience of our program, we asked a student to act as a user and attempt to operate our system. As our tester, we asked the student to document running statistics and surveyed the practicality of our tool.

Our ultimate goal is to create a very efficient and multifunctional pipeline which can not only do variant annotation, but also has multiple functional annotation databases incorporated into the pipeline. This would require downloading many databases and consistently formatting them.

In the future, we plan to add additional features/annotations to the pipeline. Some examples include population allele frequencies, functional prediction scores etc. This will be a priority for us. Additionally, we expect to compare the annotations made by SNPAAMapper with other established tools in the future version to give users a better understanding of the performance of our tool. Furthermore, to make SNPAAMapper more easily accessible to a wider range of users, we plan to extend our program to support R in future development.

## Data availability statement

The original contributions presented in the study are included in the article/Supplementary material, further inquiries can be directed to the corresponding author/s.

## Author contributions

CL, KM, and YB drafted the manuscript. NX deployed the SNPAAMapper-Python GitHub website. CF assisted to modify the pipeline codes. CL has processed the input files to generate the ClinVar results. AH conducted the student test runs. XL and YB supervised the project and provided suggestions and guidance on directions. All authors participated in the discussions and revisions. All authors contributed to the article and approved the submitted version.

## Funding

Funding for article processing charge is provided by the University of South Florida to XL.

## Conflict of interest

The authors declare that the research was conducted in the absence of any commercial or financial relationships that could be construed as a potential conflict of interest.

## Publisher's note

All claims expressed in this article are solely those of the authors and do not necessarily represent those of their affiliated organizations, or those of the publisher, the editors and the reviewers. Any product that may be evaluated in this article, or claim that may be made by its manufacturer, is not guaranteed or endorsed by the publisher.

## Supplementary material

Supplementary Figure 1 Algorithm for classifying variants by genomic regions for SNPAAMapper-Python.

Supplementary Table 1 Column description for the first 15 columns of the VCF output file.
