# Supplementary material for: SNPAAMapper-Python: A highly efficient genome-wide SNP variant analysis pipeline for Next-Generation Sequencing data
Source: Front Artif Intell. 2022 Sep 12;5:991733. doi: 10.3389/frai.2022.991733 (PMC9510352; doi:10.3389/frai.2022.991733)
Supplement: Supplementary Figure 1 — Algorithm for classifying variants by genomic regions for SNPAAMapper-Python. [file Data_Sheet_1.docx]

**Figure 1. Algorithm for Classifying variants by genomic regions for SNPAAMapper-Python**
