# Supplementary material for: SNPAAMapper-Python: A highly efficient genome-wide SNP variant analysis pipeline for Next-Generation Sequencing data
Source: Front Artif Intell. 2022 Sep 12;5:991733. doi: 10.3389/frai.2022.991733 (PMC9510352; doi:10.3389/frai.2022.991733)
Supplement: Supplementary Table 1 — Column description for the first 15 columns of the VCF output file. [file Table_1.DOCX]

| Column Name | Column Description |
| --- | --- |
| Chromosome | The first column in the VCF file, which labels the chromosome that the SNP is on. |
| Variant Position | The second column in the VCF file, which labels the coordinate of the SNP with respect to GRch37/hg19 assembly. |
| Gene Symbol | The third column in the VCF file, which labels the gene name (symbol) that the SNP is hosted on. The gene that hosts the most severe consequence made by the variant was presented as the annotated gene. |
| UCSC ID | The fourth column in the VCF file, which labels the UCSC gene ID, which is an alternative gene name. |
| Strand | The fourth column in the VCF file, which labels the strand which gene is located at. A plus strand was labelled as ‘+’ and a minus strand was labelled as ‘-’. |
| Amino Acid Position of Mutation (for CDSHIT) | The sixth column of the VCF file, which labels the Amino Acid relevant position where the mutation occurs, but only for variants located in coding sequences (CDS). |
| Variant Type | The seventh column of the VCF file, which labels the variant type (e.g. SNP or INDEL) of the detected mutation. |
| AA Ref -> AA SNP | The eighth column of the VCF file, which labels the specific Amino Acid change because the mutation occurs. Both one letter and three letter amino acid codes are provided where applicable. |
| Variant Class | The nineth column of the VCF file, which labels the specific mutation change type or the functional consequence (e.g. synonymous, non-synonymous, etc.) due to SNP happened in the coding region. Possible values are ‘NSM’ for nonsynonymous variant; ‘SYN’ for synonymous variant; ‘NSN’ for nonsense variant; ‘---‘for variants not located in CDS. |
| Ref AA Chain | The tenth column of the VCF file, which lists the reference amino acid sequence chain. |
| Alt AA Chain | The eleventh column of the VCF file, which lists the mutated amino acid sequence chain. |
| Hit Type | The twelfth column of the VCF file, which labels the gene-based annotation of the  variant. Possible values include ‘CDSHIT’, ‘UTR3’, ‘UTR5’, ‘UPSTREAM’,  ‘DOWNSTREAM’, and ‘INTRONHIT’. |
| Known dbSNP | The thirteenth column of the VCF file, which shows whether the SNP has been reported in dbSNP database or is a novel discovery. |
| Ref nt | The fourteenth column of the VCF file, which displays the reference nucleotide. |
| Alt nt | The fifteenth column of the VCF file, which displays the mutated nucleotide. |
